# Gene expression profiling of human prostate cancer stem cells reveals a pro-inflammatory phenotype and the importance of extracellular matrix interactions

**DOI:** 10.1186/gb-2008-9-5-r83

**Published:** 2008-05-20

**Authors:** Richard Birnie, Steven D Bryce, Claire Roome, Vincent Dussupt, Alastair Droop, Shona H Lang, Paul A Berry, Catherine F Hyde, John L Lewis, Michael J Stower, Norman J Maitland, Anne T Collins

**Affiliations:** 1 Pro-Cure Therapeutics Ltd, The Biocentre, Innovation Way, York Science Park, Heslington, York YO10 5NY, UK; 2 YCR Cancer Research Unit, Department of Biology, University of York, York YO10 5YW, UK; 3 Hull York Medical School, University of York, Heslington, York YO10 5DD, UK; 4 York Centre for Complex Systems Analysis, Department of Biology, University of York, York YO10 5YW, UK; 5 Department of Urology, York Hospital, Wigginton Road, York YO31 8HE, UK

## Abstract

**Background:**

The tumor-initiating capacity of many cancers is considered to reside in a small subpopulation of cells (cancer stem cells). We have previously shown that rare prostate epithelial cells with a CD133^+^/α_2_β_1_^hi ^phenotype have the properties of prostate cancer stem cells. We have compared gene expression in these cells relative to their normal and differentiated (CD133^-^/α_2_β_1_^low^) counterparts, resulting in an informative cancer stem cell gene-expression signature.

**Results:**

Cell cultures were generated from specimens of human prostate cancers (n = 12) and non-malignant control tissues (n = 7). Affymetrix gene-expression arrays were used to analyze total cell RNA from sorted cell populations, and expression changes were selectively validated by quantitative RT-PCR, flow cytometry and immunocytochemistry. Differential expression of multiple genes associated with inflammation, cellular adhesion, and metastasis was observed. Functional studies, using an inhibitor of nuclear factor κB (NF-κB), revealed preferential targeting of the cancer stem cell and progenitor population for apoptosis whilst sparing normal stem cells. NF-κB is a major factor controlling the ability of tumor cells to resist apoptosis and provides an attractive target for new chemopreventative and chemotherapeutic approaches.

**Conclusion:**

We describe an expression signature of 581 genes whose levels are significantly different in prostate cancer stem cells. Functional annotation of this signature identified the JAK-STAT pathway and focal adhesion signaling as key processes in the biology of cancer stem cells.

## Abstract 2

An expression signature of human prostate cancer stem cells identifies 581 differentially expressed genes and suggests that the JAK-STAT pathway and focal adhesion signaling are important.

## Background

The concept of a cancer stem cell within a more differentiated tumor mass, as an aberrant form of normal differentiation, is now gaining acceptance over the current stochastic model of oncogenesis, in which all tumor cells are equivalent both in growth and tumor-initiating capacity [1,2]. For example, in leukaemia, the ability to initiate new tumor growth resides in a rare phenotypically distinct subset of tumor cells [3] that are defined by the expression of CD34^+^CD38^- ^surface antigens and have been termed leukemic stem cells. Similar tumor-initiating cells have also been found in 'solid' cancers, such as prostate [4], breast [5], brain [6], lung [7] colon [8,9] and gastric cancers [10]. We have recently shown that a rare cell population in human prostate cancer, defined by the phenotype CD133^+^/α_2_β_1_^hi ^(high expression of α_2_β_1 _integrin) and comprising less than 0.1% of the tumor mass, has many of the properties of cancer stem cells [4]. In particular, self renewal, extended lifespan (compared to normal stem cells), a high invasive capacity, a primitive epithelial phenotype and an ability to differentiate to recapitulate the phenotypes seen in prostate tumors. The cancer stem cell content was not, however, dependent on prostate tumor clinical stage or grade.

Numerous groups have profiled prostate cancer using DNA microarrays (reviewed in [11]). Despite this, the genetic changes associated with initiation and progression of this disease remains undefined. Traditionally, expression profiling has focused on sampling the tumor cell mass, but this does not take into account the genetic and phenotypic heterogeneity of tumors. Moreover, individual genes are identified rather than sets of genes that share a biological function. Here we report the first expression profile of a stem cell population from human prostate cancers. By further analyzing this expression signature in the context of biological function, key pathways have been identified that are associated with inflammation, extracellular matrix interactions and stem cell self-renewal.

## Results

### Identification of gene products associated with a cancer stem cell phenotype

**Figure 1:**
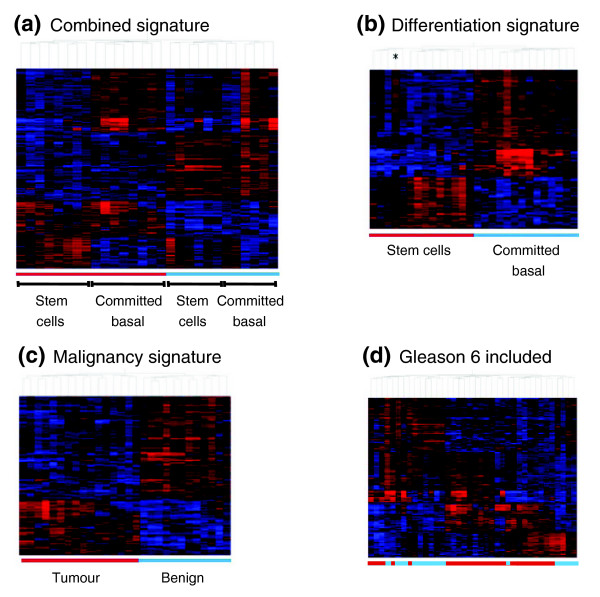
Distinctive stem cell and tumor signatures are found in human prostate cancers containing a minimum Gleason score 7 pathology. Clustering analysis (derived from the Pearson correlation) using the expression data for the probesets (from 28 samples) define a cancer stem cell signature. Blue tiles indicate down-regulated genes, and red tiles indicate up-regulated genes. **(a) **The combined signature clustered samples as benign (blue bar) and malignant (red bar). Cell type (stem, CD133^+^/α_2_β_1_^hi^; and committed, CD133^-^/α_2_β_1_^low^) was also defined within each disease state. **(b) **The differentiation signature. One sample in which a clear differentiation signature 'breakthrough' was evident in the combined signature is indicted by an asterisk. **(c) **Sample clustering according to the malignancy signature. **(d) **Hierarchical clustering with the Gleason 6 samples and a single hormone treated sample included in the analysis. Note that the clear distinction between non-malignant and malignant biopsies is lost by including this data.

By comparing RNA expression patterns from stem and committed cells, independent of their disease status, 287 probesets showed significantly elevated expression in stem cells (Welch *t *test, *p *< 0.035). Comparison of the expression patterns from normal stem cells with those from malignant stem cells (Gleason score >7) identified 333 probesets with significantly increased expression in malignant cells. (Welch *t* test, *p *< 0.1). These were combined to give a 620 probeset 'cancer stem cell signature'. The occurrence of multiple probes for the same gene in our dataset gave us a final signature of 581 genes when we translated probe IDs to gene names. We used hierarchical clustering to demonstrate that the genes identified in our cancer stem cell signature could be used to distinguish between different phenotypic groups within our data set. The combined cancer stem cell signature successfully separated benign from malignant samples. Within the different disease states we found that samples with the same differentiation state clustered together (Figure 1a). Using the separated differentiation and malignancy signatures we were able to cluster samples according to their differentiation or disease states, respectively (Figure 1b,c). However, if data from Gleason 6 tumors or a single Gleason 7 patient, on hormone-deprivation therapy, were included in the clustering analysis, then we were unable to distinguish between benign and malignant samples, as well as differentiation state (Figure 1d). For this reason Gleason 6 samples and hormone refractory samples were excluded from subsequent analyses. We also noted that in one stem cell sample a clear differentiation signature was evident (Figure 1b, asterisk), which was most likely due to contamination of the CD133^+^/α_2_β_1_^hi ^fraction with more differentiated cells.

**Figure 2:**
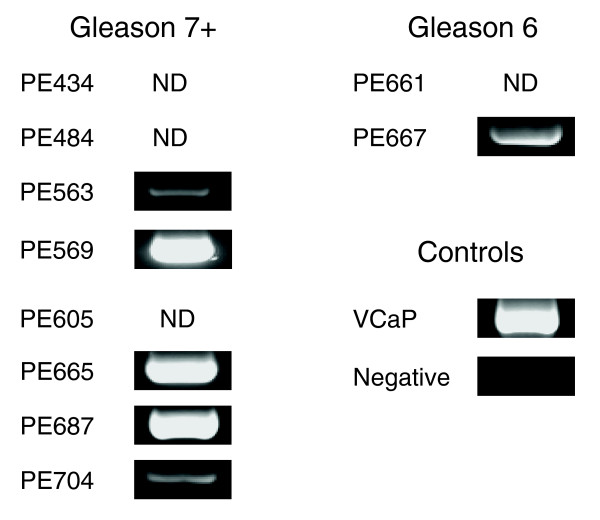
Nested RT-PCR for the detection of the *TMPRSS2:ERG *fusion. Samples from the microarray data set, where sufficient material was available, were subjected to nested RT-PCR to detect the presence of the TMPRSS2:ERG fusion product. The fusion product was detected in 6 of 10 samples and undetectable in the remainder (samples marked ND). cDNA from the fusion positive cell line VCaP was used as a positive control, water was substituted in place of cDNA for the negative control.

Although there was a clear distinction between malignant and benign samples we used an RT-PCR based approach to screen for the presence of the fusion transcript *TMPRSS2*:*ERG *(transmembrane protease, serine 2:v-ets erythroblastosis virus E26 oncogene homolog fusion product) as a further test for tumorigenicity [12]. We found that 62% or 5 out of 8 cultures (Gleason score 7 and above) expressed *TMPRSS2*:*ERG *(Figure 2). Interestingly, a culture derived from a lymph node metastasis of the prostate did not express the transcript (PE704), yet expression was detected in one culture derived from a Gleason 6 tumor.

### Cancer stem cells express known prostate cancer-associated genes

**Figure 3:**
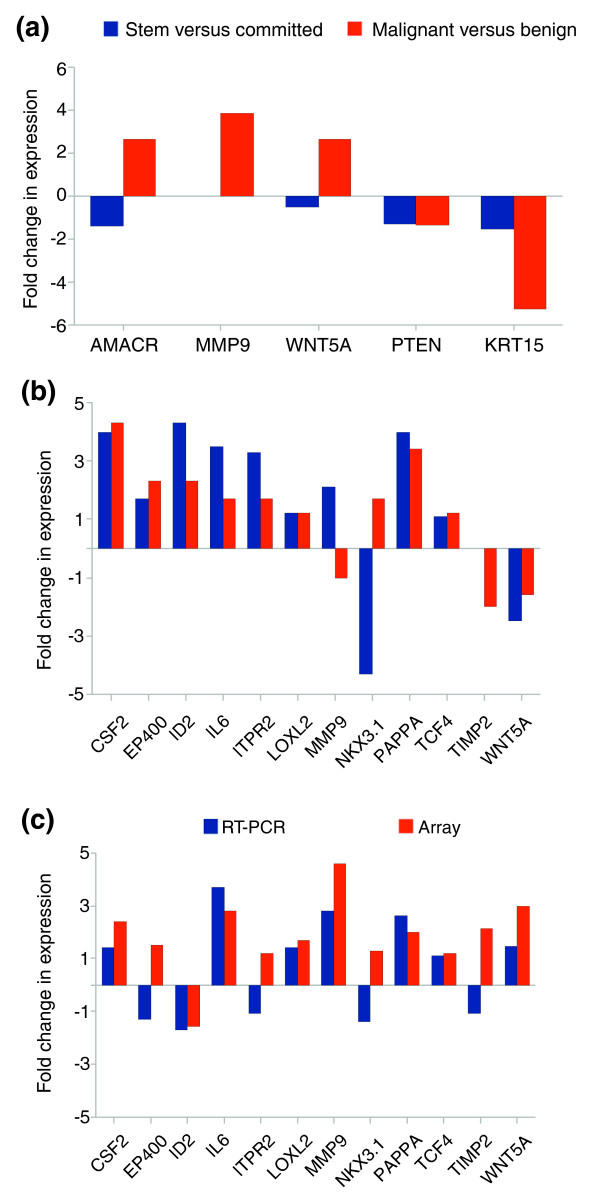
Validation of selected genes by quantitative real time PCR. **(a) **RT-PCR confirmation of Affymetrix array data on genes associated with prostate cancer (all changes in expression were significant at *p *< 0.05). Changes between stem and committed cells are indicated in blue, while malignant versus benign changes are indicated in red. **(b) **Validation of average changes in gene expression between stem and committed basal populations detected by Affymetrix array (red bars) and RT-PCR techniques (blue bars). **(c) **Validation of average changes in gene expression between malignant and benign stem cell populations detected by Affymetrix (red bars) and RT-PCR techniques (blue bars).

The cancer phenotype was validated by confirming the expression levels of several established prostate cancer markers from the Affymetrix dataset by real time PCR (Figure 3a). For example, alpha-methylacyl-CoA racemase, a phenotypic marker identified in the first microarray experiments on prostate cancer [13], was significantly over-expressed in malignant samples, but under-expressed in stem cells relative to committed cells. Similarly, matrix metalloproteinase (MMP)9 and WNT5A were also over-expressed in malignant samples, but not in the stem cell population. As expected, PTEN (phosphatase and tensin homolog) showed a modest down-regulation in malignant and stem populations as did Cytokeratin-15, which has been shown to be associated with the benign prostatic hyperplasia (BPH) cell type [14].

A panel of genes was selected to confirm the reproducibility of the array data by real time PCR. Comparison of stem versus committed populations demonstrated expression changes in the same direction as the array data, in 10 out of the 12 genes studied (83%), but variations in magnitude were observed (Figure 3b). Similar results were obtained when comparing benign and malignant samples, although some genes (4 out of 12; 33%) did display inconsistencies between microarray and PCR assays (Figure 3c).

### Gene expression signature associated with the cancer stem cell phenotype

Following the definition of the cancer stem cell signature, we proceeded to explore the different functional groups present in the dataset. Genes associated with inflammation were particularly prominent in this set of over-expression products. In particular, nuclear factor κB (NF-κB) and interleukin (IL)6 were up-regulated in the cancer stem cell population, as were multiple genes associated with cell-cell communication and adhesion (for example tight junction protein (TJP)2/ZO2 and integrin alpha V). The gene showing the highest differential expression in the cancer stem cell population, by up to four-fold (Table 1 and Figure 3b), was that encoding the secreted metallo-protease Pappalysin A (PAPPA) [15].

**Table 1:** Candidate genes whose expression is altered in the cancer stem cell population

Gene description	Symbol	Stem versus committed*	Malignant versus benign*
Pregnancy-associated plasma protein A, pappalysin 1	*PAPPA*	3.83	3.26
Nuclear factor of kappa light polypeptide gene enhancer in B-cells 1 (p105)	*NFKB1*	2.30	1.86
Tight junction protein 2 (zona occludens 2)	*TJP2*	2.51	1.72
Abl-interactor 1	*ABI1*	3.41	1.84
B-cell translocation gene 1, anti-proliferative	*BTG1*	3.95	1.53
Interleukin 6 (interferon, beta 2)	*IL6*	1.93	5.18
CASP8 and FADD-like apoptosis regulator	*CFLAR*	1.90	1.41
Smu-1 suppressor of mec-8 and unc-52 homolog (*C. elegans*)	*SMU1*	1.90	1.63
S100 calcium binding protein A3	S100A3	1.92	1.55
Chromosome 17 open reading frame 27	C17orf27	1.63	1.80
RAS and EF-hand domain containing	*RASEF*	2.31	1.71
Integrin, alpha V (vitronectin receptor, alpha polypeptide, antigen CD51)	*ITGAV*	2.55	1.62
Interferon gamma receptor 1	*IFNGR1*	1.70	1.46
			
Insulin growth factor-like family member 1	*IGFL1*	-1.17	-28.61
Microseminoprotein, beta- (PSP94)	*MSMB*	-27.78	-2.84
Prostate stem cell antigen	*PSCA*	-20.11	-2.27
Carcinoembryonic antigen-related cell adhesion molecule 5	*CEACAM5*	-19.12	-1.77
S100 calcium binding protein A7 (psoriasin 1)	S100A7	-11.48	2.28
Hydroxyprostaglandin dehydrogenase 15-(NAD)	*HPGD*	-8.54	-2.41
Carcinoembryonic antigen-related cell adhesion molecule 7	*CEACAM7*	-19.22	2.95
Trefoil factor 1 (breast cancer, estrogen-inducible sequence expressed in)	*TFF1*	-11.74	-2.25
Prolactin-induced protein	*PIP*	-17.42	1.04

*Values are mean fold expression changes abstracted from Affymetrix datasets. Positive values (top half) indicate over expression in the cancer stem cell samples. Negative values (bottom half) indicate genes over-expressed in the committed cell or benign cell fractions.

**Figure 4:**
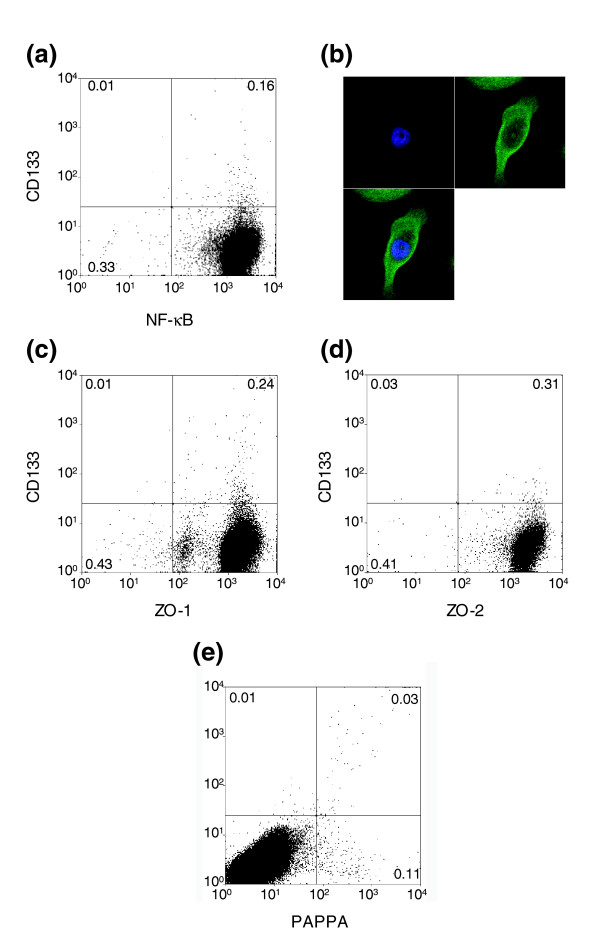
Validation of selected genes by flow cytometry and immunocytochemistry. **(a) **Flow cytometry analysis of prostate cancer cells co-stained with antibodies to CD133 and the NF-κB p65 subunit. **(b) **Confocal image of sorted CD133^+ ^cancer cells stained with an antibody to the NF-κB p65 subunit (green) counterstained with DAPI (blue). Nuclear concurrence of two signals is indicated by a cyan colour. **(c-e) **Flow cytometry analysis of prostate cancer cells co-stained with antibodies to CD133 and ZO1/TJP1 (c) or ZO2/TJP2 (d) or PAPPA (e).

Further validation of differential expression was carried out at the protein level using a combination of flow cytometry and immunocytochemistry (Figure 4). Using antibodies to CD133 and NF-κB on primary tumor cultures demonstrated that both progenitor and stem cells expressed NF-κB protein (Figure 4a). Nuclear localization of NF-κB was evident by immunocytochemistry on CD133-selected tumor cells treated with tumor necrosis factor (TNF)α (Figure 4b). This confirmed that the active form of the protein was present in the stem cell population. TJP1 (ZO-1) and TJP2 (ZO-2) proteins were also expressed by the majority of progenitor and stem cells from tumor cell cultures (Figure 4c,d), whereas only a minority of the total cell population expressed PAPPA (Figure 4e). Nevertheless, this protein was present in a majority of the CD133^+^/α_2_β_1_^hi ^population.

### Parthenolide treatment affects cancer stem cells but not normal progenitor and stem cell activity

**Figure 5:**
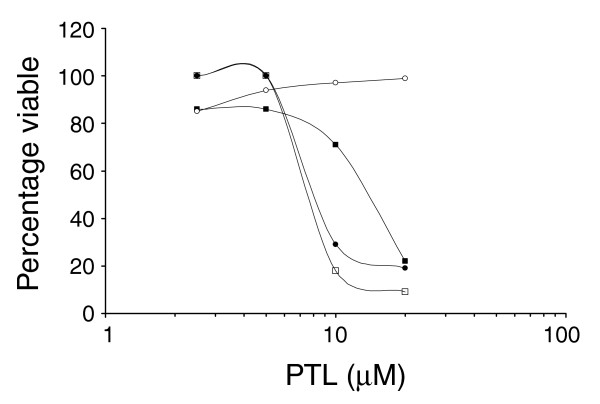
PTL induces apoptosis in primitive cancer cells. Percent viability of prostate cancer cells and cells from a patient with BPH treated with increasing concentrations of PTL. Cells were cultured for 1 h with 100 ng/ml TNFα prior to treatment with PTL for 18 h. Cells were subsequently labeled with CD133-APC, Annexin-V-FITC and DAPI. Viability was defined as annexin-V^-^/DAPI^- ^on total cells. Three prostate cancer patients' samples were analyzed and a representative profile is shown of normal CD133^+ ^(open circles), cancer CD133^+ ^(filled squares), normal progenitor (filled circles) and cancer progenitor (open squares).

To functionally assess the effects of blocking NF-κB signaling, cells were treated with the sesquiterpene lactone parthenolide (PTL). As NF-κB is known to promote cell survival [16], we determined whether its inhibition by PTL could preferentially induce cell death in primary tumor cells while sparing normal cells. Figure 5 shows an example of annexin V staining of cancer and normal prostate cells in response to an 18 hour treatment with PTL. Although normal CD133^+ ^cells show almost no loss of viability in the presence of PTL, the cancer CD133^+ ^cells were strongly induced to undergo apoptosis (from 88% to 22% viability after treatment) as were the progenitor cells from cancer and normal cultures.

### Functional annotation of the cancer stem cell signature

**Figure 6:**
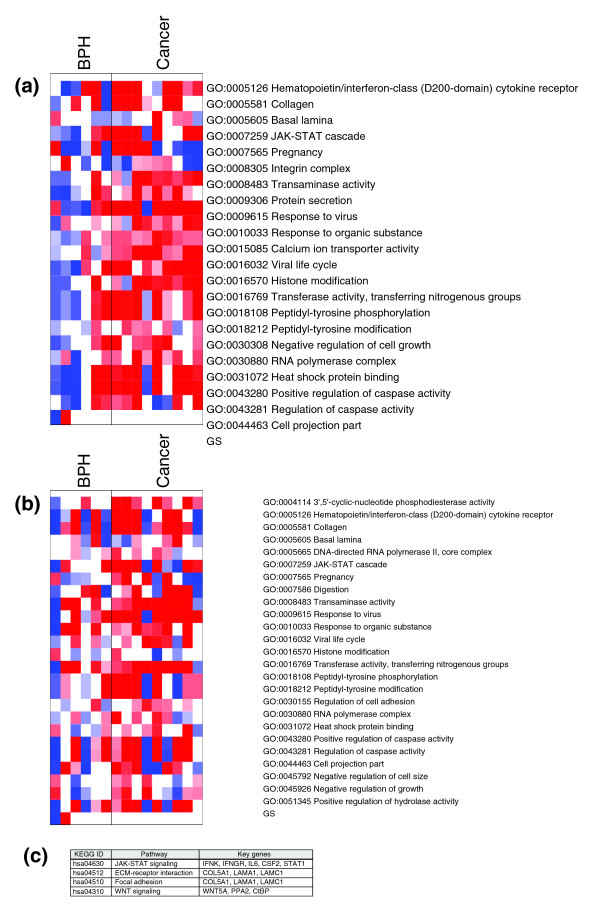
Functional annotation of the cancer stem cell expression signature. **(a,b)** Functional concepts over-represented in cancer relative to BPH within the stem cell population (a) or within the committed basal population (b) derived from the GO. Over-represented terms are shown in red, and under-represented terms are shown in blue. **(c) **Examples of key pathways and related genes involved in over represented gene ontology functions.

We used annotation data from the Gene Ontology (GO) [17] to identify key functional categories within the gene expression signature. The cancer stem cell signature was subjected to gene set enrichment analysis (GSEA) to identify over-represented GO terms [18]. We identified 22 GO terms that were significantly over-represented (*p *< 0.01) in cancer samples within the stem cell population (Figure 6a) and 25 GO terms significantly over-represented (*p *< 0.01) in cancer samples within the committed basal population (Figure 6b). We found 17 functional concepts that were common to both stem and committed basal populations. Mapping these 17 GO terms against our cancer stem cell signature identified 28 genes. Searching these 28 genes against the Kyoto Encyclopedia of Genes and Genomes (KEGG) pathway database [19] highlighted 4 main pathways (Figure 6c). These pathways were dominated by the signaling of inflammatory cytokines through the JAK-STAT (Janus activated kinase-signal transducer and activator of transcription) pathway and the interaction of cell surface receptors with the extracellular matrix and associated downstream signaling. Our cancer stem cell signature also contained several other genes that might reasonably be considered part of this system, but are not currently annotated to known pathways in the KEGG database [19], for example, those encoding collagens 8A1, 12A1, 16A1 and 27A1.

We then extended our search to look for components of these pathways that were present in our gene expression signature, but were not identified by GSEA. This search returned a total of 8 members of the JAK-STAT pathway, 7 components of the extracellular matrix-receptor system and 15 components of the focal adhesion signaling pathway. It is worth noting that five members of the focal adhesion pathway and the extracellular matrix-receptor system overlap, as the focal adhesion pathway is activated by extracellular matrix-receptor interaction.

## Discussion

Despite advances in both screening and in surgical treatment, the long-term prognosis for patients with hormone relapsed prostate cancer remains disappointingly poor [20]. Current tumor targeting strategies for therapy are largely based on differentiation antigens, such as prostate specific antigen and androgen receptor, but our previous studies have shown that the cells that self-renew are a population of primitive cells with the phenotype α_2_β_1_^hi^/CD133^+^, which are most likely unaffected by current chemotherapeutic regimes [4]. Accordingly, previous expression array studies of prostate have been dominated by androgen receptor-regulated gene products derived from more abundant differentiated cells and the higher average gene expression in these cells is likely to have masked more subtle expression changes in rare cancer stem cells.

Recent advances in microarray technology and target labeling methods have opened up the possibility of performing whole genome transcription profiling experiments from small amounts of starting material, such as rare stem cells [21]. Dumur and colleagues [21] showed that the GeneChip Two-cycle sample labeling method produced similar results to the standard One-cycle method on 11 out of 12 quality control parameters tested. There was a small bias in the 3'/5' ratio of some genes caused by the generation of shorter products from the Two-cycle labeling method. However, hierarchical clustering showed that each Two-cycle labeled sample was most closely associated with its One-cycle counterpart.

The most striking conclusion from studying highly purified subpopulations from human prostate cancers was the ability of the combined tumor/differentiation cancer stem cell 'signature' to distinguish benign epithelium from tumors with a Gleason 4 morphology [22]. Interestingly, not all Gleason score 7+ cultures expressed the *TMPRSS2*:*ERG *fusion [12], including one lymph node metastasis, yet they clearly clustered away from Gleason 6 cultures (one of which expressed *TMPRSS2*:*ERG*). Recently, expression array analysis of micro-dissected prostate tumors has confirmed the hypothesis that the transition to Gleason pattern 4 is associated with significant shifts in gene expression patterns [23]. Lymph node metastases segregated with primary tumors based on the expression signature, but preliminary results indicated that hormone-refractory tumors form a distinct (and possibly more heterogeneous) subgroup in terms of gene expression, as do the Gleason 6 tumors. As the *TMPRSS2:ERG *gene fusion was detected in one out of two Gleason 6 cultures tested, and is associated with lethal prostate cancer [24], further study of larger samples of prostate cancer stem cells from different classes of therapy-resistant and Gleason 6 tumors is warranted.

Despite short-term culturing, to expand the stem cell population, the cancer signature was validated by confirming the expression levels of several established prostate cancer markers. Alpha-methylacyl-CoA racemase, a phenotypic marker identified in the first microarray experiments on prostate cancer [13], was significantly over-expressed in cancer samples, as was MMP9. High MMP expression is consistent with matrix degradation and high invasive capacity previously reported in cancer stem cell cultures [4]. As expected, PTEN showed a modest down-regulation in malignant and stem populations, consistent with the haplo-insufficiency proposed on the basis of transgenic mouse experiments [25] and in recent studies of hematopoetic tumor stem cells [26].

Several studies have investigated the differences in gene expression profiles between samples isolated directly from tissue and those from cells cultured *in vitro *[27-29]. Wick *et al*. [28] compared transcriptional profiles from *ex vivo *and *in vitro *cultured samples of human dermal lymphatic endothelial cells and blood endothelial cells. These authors found that 2.1% and 4.0% of transcripts were affected by culture in lymphatic endothelial cells and blood endothelial cells, respectively. It is worth noting that this study employed different labeling methods for *in vitro *and *ex vivo *samples, which may partially account for the discrepancy. A similar study on hepatic stellate cells highlighted the importance of culture microenvironment and the appropriate use of feeder cells in co-culture. Comparison of transcriptional profiles from hepatic stellate cells cultured *in vitro *or from cells isolated directly from tissues found substantial differences in the lists of genes found to be differentially expressed. It was shown that co-culture of hepatic stellate cells with Kupffer cells *in vitro *(acting as feeders) shifted the gene expression profile to a pattern that was consistent with that found *in vivo *[29]. This suggests that the use of feeder cells in our cultures of cancer stem cells is likely to be important for maintaining gene expression patterns similar to cancer stem cells *in vivo*.

Expression of multiple genes associated with cell-cell communication and adhesion was associated with the cancer stem cell population. These expression products have been implicated in tissue integrity [30] and the normal stem cell 'niche' [31,32]. The gene showing the highest differential expression in the cancer stem cell population was that encoding PAPPA [15]. This pregnancy-associated plasma protein specifically cleaves insulin-like growth factor binding protein (IGFBP)-4 and IGFBP-5. Proteolysis of IGFBPs regulates the bioavailability of IGFs, and because of the association between IGF levels and prostate cancer [33], strategies for the direct inhibition of IGF signaling, by inhibiting proteolytic activity, is a potential therapeutic strategy and would likely not interfere with insulin signaling [34].

We used a panel of genes, based on their known association with prostate biology and cancer, to confirm the reproducibility of the array data. Most genes were consistent, but we did note discrepancies, particularly between the malignant and benign RT-PCR results, which may be due to patient variability. In all cases where discrepancy exists, the fold change in expression as measured by RT-PCR was less than two and these small differences are difficult to reproduce accurately. In some cases the absolute expression levels of the genes were quite low, which makes them more sensitive to small fluctuations. The discrepancy could also be caused by the use of probes targeted to different regions of the transcript. Real-time PCR probes are commonly designed against the consensus sequence of the known transcripts for the target gene. Microarrays carry multiple probes against the same gene distributed throughout the length of the transcript, some of which detect only a subset of the known transcripts for the target gene.

Despite this, our data suggest that the transcription factor NF-κB may be a promising therapeutic target as PTL, which acts directly on NF-κB and prevents it entering the nucleus, appeared to promote selective cell death of the cancer-specific CD133 population. Similar results have been demonstrated for leukemic CD34^+^ stem cells, with normal CD34 cells spared from apoptosis [35].

Functional annotation of the cancer stem cell signature by GSEA led us to four main pathways: JAK-STAT signaling; cell adhesion and extracellular matrix-interactions; focal adhesion signaling; and WNT signaling. There is a substantial body of work linking Wnt signaling with stemness and malignant behavior (reviewed in [36]). With respect to prostate cancer, Wnt signaling has been linked to progression to androgen-independence and bone metastasis [37,38].

Extracellular matrix-receptor signaling and the focal adhesion pathway can be considered part of the same system, as the focal adhesion pathway is activated by extracellular matrix-receptor interaction. Changes in extracellular matrix and associated proteins have been reported in the metastatic progression of prostate cancer [39], and activation of Focal adhesion kinase through α_5_β_1 _integrin/fibronectin has previously been implicated in regulating the invasiveness of prostate cancer cells via activation of phosphatidylinositol-3,4,5-trisphosphate kinase [40]. The JAK-STAT pathway could also be considered to overlap with this system since focal adhesion signaling, as defined in the KEGG database, can be activated by cytokine-cytokine receptor interaction, which is also the major activation method of the JAK-STAT pathway. In addition, JAK-STAT and focal adhesion signaling share several common components, such as the GRB-SOS (growth factor receptor-bound protein 2-son of sevenless) complex and the phosphatidylinositol-3,4,5-trisphosphate kinase/Akt axis. The involvement of IL6 and the JAK-STAT pathway in advanced prostate cancer is well known [41,42]. More recently, STAT1 has emerged as a potential mediator of drug resistance in prostate cancer [43] and may present a potential therapeutic target.

## Conclusion

Our ability to select and culture stem cell populations will now allow us to determine the genotype of these cells for permanent (mutagenic) changes, such as characteristic translocations [12] and the presence of epigenetic control [44]. We should also now be able to monitor the effects of novel therapeutics on the cancer stem cell population. Advances in viable cell separation technology and the first detailed expression signature reported here now provide the means to update and ultimately test the cancer stem cell hypothesis in a common non-hematological tumor.

## Materials and methods

### Tissue collection, isolation, and culture of tumor stem cells

Human prostate tissue was obtained, with patient consent, from 12 patients undergoing radical prostatectomy and transurethral resection for prostate cancer and 7 patients undergoing transurethral resection of the prostate for benign prostatic hyperplasia (age range 52-79 years; Table 2). Prostate cancer was confirmed by: histological examination of representative adjacent fragments; *in vitro *invasion [4]; and expression of the fusion product *TMPSS2*:*ERG *[12] (Figure 2). To preclude the need for extensive enzymatic amplification cycles prior to Affymetrix analysis, cultures were generated from isolated stem cells (CD133^+^/α_2_β_1_^hi^), as described previously [4]. In some cases, cultures were derived initially from the more abundant α_2_β_1_^hi ^population (which contains the CD133^+ ^fraction), usually from small biopsies (lymph node metastasis and core biopsies of the prostate).

**Table 2:** Summary of patient population and invasive characteristics of corresponding stem cell cultures *in vitro*

Patient number	Age (years)	Origin	Gleason score	% Invasion in vitro*
228	-	LN metastasis	7	101 ± 21
434	59	Prostate	8/9	99 ± 56
484	69	Prostate	7	105 ± 29
512	74	Prostate	BPH	-
561	72	Prostate	BPH	-
563	64	Prostate	7	35 ± 9.5
569	64	Prostate	8	75 ± 9
574	74	Prostate	BPH/G6 (5%)	-
605	56	LN metastasis	7	119 ± 21
661	78	Prostate	6	-
627	79	Prostate	BPH	-
662	66	Prostate	BPH	-
665	53	Prostate	7	63 ± 18.4
667	47	Prostate	6	61 ± 4.2
687	63	Prostate	7	74
690	79	Prostate	BPH	-
693	75	Prostate	BPH	-
704	64	Prostate	7 (HR)	-
003/06	52	Prostate	6	-

*Invasion assays were carried out on total epithelial cell populations before fractionation according to [4]. Positive controls for invasive capacity were cell lines MCF7 and PC3M whose invasion score was 18-36%, whereas normal cell lines PNT2 and PNT1a and BPH/primary normal prostate invasion scores ranged from 3-6%. Patient 704 was being treated (hormone refractory (HR)) by androgen blockade therapy.

### Nested RT-PCR for the detection of the *TMPRSS2:ERG *fusion

RNA was extracted from prostate tissue using the Qiagen RNeasy kit (Qiagen, Crawley, UK) following the manufacturer's instructions. The RNA was reverse transcribed using random hexamers and reverse transcriptase (Superscript III, Invitrogen, Paisley, UK).

Specific primers were used to detect the presence of the *TMPRSS2:ERG *fusion by nested RT-PCR (first step, forward 5'-CGC GAG CTA AGC AGG AGG C-3' and reverse 5'-GGC GTT GTA GCT GGG GGT GAG-3'; 2nd step, forward 5'-GGA GCG CCG CCT GGA G-3' and reverse 5'-CCA TAT TCT TTC ACC GCC CAC TCC-3'; Invitrogen). Each PCR reaction contained 1 μM of the respective forward and reverse primers, 1.5 mM MgCl_2_, 0.2 mM dNTPs and 1 U Taq polymerase (GoTaq, Promega, Southampton, UK). The PCR conditions were adapted from those of Clarke *et al*. [45]. Briefly, the first step PCR conditions were 94°C for 30 s followed by 35 cycles of 94°C for 20 s and an extension step of 68°C for 1 minute. There was no annealing step as the region amplified is very GC rich. The second step conditions were 94°C for 30 s, 35 cycles of 94°C for 20 s, 66°C for 10 s and 68°C for 1 minute followed by 68°C for 7 minutes.

PCR products were separated by electrophoresis through a 1.5% agarose GelRed (Invitrogen) stained gel for 1 h at 80 V. PCR products were visualized using a Gene Genius bio-imaging system.

### Array sample and data processing

#### Total RNA extraction

Total RNA was extracted from up to 1 × 10^4 ^CD133^+^/α_2_β_1_^hi ^selected cells from malignant and non-malignant cultures using Qiagen RNeasy micro-columns according to the manufacturer's protocol. For CD133^-^/α_2_β_1_^low ^cells, total RNA was extracted from between 1 × 10^5 ^and 1 × 10^6 ^selected cells using Qiagen RNeasy mini-columns. RNA yields were determined spectrophotometrically at 260 nm and RNA integrity checked by capillary electrophoresis using an Agilent 2100 bioanalyzer (Agilent, South Queensferry, UK).

#### Production of fragmented labeled cRNA

Total RNA (10-50 ng) was amplified using two rounds of cDNA synthesis and *in vitro *transcription, and biotin labeled by following the Affymetrix small scale labeling protocol VII [46], omitting the T4 DNA polymerase steps in the two second strand cDNA synthesis reactions and using the Affymetrix GeneChip *in vitro *transcription labeling kit for the second cycle *in vitro *transcription for cRNA amplification and labeling. The Affymetrix eukaryotic sample and array processing standard protocol was followed at this stage and the quality of first and second round cRNA products and fragmented cRNA was checked by capillary electrophoresis using an Agilent 2100 bioanalyzer.

#### Array hybridization

Labeled fragmented cRNA (10 μg) was hybridized to oligonucleotide probes on an Affymetrix HG-U133plus2 GeneChip, according to the hybridization, washing, staining and scanning procedure in the Affymetrix eukaryotic sample and array processing standard protocol (Affymetrix Fluidics Station 450 using the EukGE-WS2v5 protocol). Final scanning of the arrays was carried out with an Affymetrix Gene Scanner 3000. The raw data are available in the ArrayExpress Database (accession E-MEXP-993).

#### Data processing

Scanned GeneChip images were processed using Affymetrix GCOS 1.2 software to derive an intensity value and flag (present, marginal or absent) for each probe. Probe intensities were derived using the MAS5.0 algorithm. Comparisons between different sample datasets were conducted using Agilent GeneSpring GX software. Datasets to be compared were first normalized using three steps (consecutively applied in the order given): by transforming values <0.01 to 0.01; normalizing each chip to the median of the measurements taken for that chip; and finally normalizing each probe to the median of the measurements for that probe. Low quality or uninformative data were removed using three selections (consecutively applied as follows): probes flagged 'absent' in all samples; probes with standard deviation within a parameter class of >1 in at least three of the four conditions; and probes with less than a two-fold overall change in normalized expression value between all four of the conditions.

### Statistical analysis

The gene expression profile of CD133^+^/α_2_β_1_^hi^ and CD133^-^/α_2_β_1_^low^ prostate cancer cells were compared with benign CD133^+^/α_2_β_1_^hi ^and CD133^-^/α_2_β_1_^low ^prostate epithelial cells. Statistical analysis of the transcription profiles was derived from patients with Gleason score 7 cancers and above. Gleason score 6 biopsies, and one Gleason score 7 biopsy (from a patient who had received hormone therapy) were excluded (Table 1).

Following removal of low quality or uninformative data (see 'Data processing') samples were subjected to a two-way ANOVA test to identify significant (*p *< 0.05) changes between malignant and benign populations and between stem (CD133^+^/α_2_β_1_^hi^) and committed (CD133^-^/α_2_β_1_^low^) populations. Gene expression changes in benign versus malignant cells (within the stem cell population) was compared using a Welch *t *test. A second Welch *t *test was used to compare stem and committed populations independent of their disease status. To define signature probesets for the cancer stem cell population, the Benjamini and Hochberg false discovery rate multiple testing correction was applied to the results of Welch *t *tests between the cell populations, resulting in a corrected critical value of *p *< 0.035. This value was used in the comparison of stem and committed populations, independent of their disease status, to define a stem cell-specific expression signature. When comparing malignant against benign samples very few probesets were significantly different at *p *< 0.035, resulting in a combined cell type/malignancy signature that was biased in favor of cell differentiation characteristics. To compensate for this, the critical value was adjusted to *p *< 0.1 for the comparison of benign and malignant components within the stem cell population. Those genes found to be significantly over-expressed in stem cells were combined with genes significantly over-expressed in malignant samples to generate a malignant stem cell signature.

### Quantitative reverse transcriptase PCR

Reverse transcription was carried out on cDNA generated from 50 ng of fractionated cell RNA purified as described above. This was either prepared freshly from RNA or taken from the second round cDNA synthesis for Affymetrix arrays (see above) where starting material was limiting. cDNA generated from the cell lines P4E6 [47] and PC346C (kindly provided by Nefkens Institute, Erasmus University, Rotterdam) was combined in a 1:1 ratio and used to generate the standard curve for each assay. Real time PCR was carried out using TaqMan gene expression pre-synthesized reagents and master mix (Applied Biosystems, Warrington, UK). Reactions were prepared following the manufacturers protocol except that a reduced total volume of 25 μl was used. All reactions were carried out in triplicate on 96-well PCR plates (ABgene, Epsom, UK) in an ABI PRISM 7000 sequence detection system (Applied Biosystems). Standard thermal cycling conditions included a hot start of 5 minutes at 50°C, 10 minutes at 95°C, followed by up to 50 cycles of: 95°C 15 s, 60°C for 1 minute. Data analysis was carried out using ABI SDS software and Microsoft Excel. Expression values are presented relative to the geometric mean of the measurements for three endogenous control genes (*GAPDH*, *ITGB1 *and *PPIA*) in the corresponding samples.

### Functional annotation of the prostate cancer stem cell signature

Genes found to be differentially expressed were analyzed for over representation of GO terms to identify important functional categories for further study [17]. Analysis was performed using the PGSEA package for the R environment available through the Bioconductor project [18,48,49]. Our analysis was designed to identify GO terms that were significantly over-represented (*p *< 0.01) in cancer versus benign samples within the stem cell population or within the committed population. We then mapped significant GO terms back to the cancer stem cell signature to identify the individual genes involved. These genes were then searched against the KEGG pathway database [19] to identify the critical pathways.

### Validation by immunocytochemistry and flow cytometry

CD133^+^/α_2_β_1_^hi ^cells were selected from cultured cells before processing for dual-color imaging under confocal microscopy by fixation in a 50:50 mix of ice-cold methanol/acetone or 4% paraformaldehyde in phosphate-buffered saline. After blocking with 20% normal serum in Tris buffered saline, cells were incubated with monoclonal antibodies against the NF-κB p65 subunit (Chemicon International, Hampshire, UK) or a non-specfic isotype control. Appropriate positive control cells were stained in parallel for each antibody. After washing (3 × Tris buffered saline), cells were labeled with Alexa Fluor^® ^488-tagged secondary antibody (Invitrogen). Cells were mounted in the anti-photobleaching medium Vectashield containing 4',6-diamino-2-phenylindole (DAPI; Vector Laboratories, Peterborough, UK). Cultured cells were processed for dual-color staining flow cytometry as described previously [4]. Cells were co-stained with CD133 (clone 293C; Miltenyi Biotec Ltd, Bisley, UK) and antibodies to Pappalysin 1A (a kind gift from Dr Claus Oxvig, University of Aarhus, Denmark) or ZO-1 (clone ZO1-1A12), ZO-2 (clone 3E8D9; Zymed Laboratories Inc., San Franscisco, CA, USA) and the NF-κB p65 subunit (Chemicon International). Cells were separated on a DakoCytomation CyAn high-performance flow cytometer and analyzed using DakoCytomation Summit version 3.3 software.

### Apoptosis assay

Unselected cells were treated for 18 h with increasing concentrations of PTL in the presence of TNFα. Cells were subsequently stained with anti-CD133-APC (anti-CD133-allophycocyanin; Miltenyi Biotec Ltd) for 10 minutes on ice. Cells were then washed in cold magnetic assisted cell sorting (MACS) buffer and resuspended in annexin binding buffer (10 mM HEPES/NaOH, pH 7.4, 140 mM NaCl, 2.5 mM CaCl_2_). Annexin V-FITC (Pharmingen, Oxford, UK) and 0.25 μg/ml DAPI were then added for 15 minutes before analysis by flow cytometry. The percent viable cells was defined as annexin-V^-^/DAPI^- ^cells on total (ungated) cells and on gates set for CD133^+ ^populations. The total number of events collected was between 1 × 10^5 ^to 1 × 10^6 ^depending on the CD133 content of the sample.

## Abbreviations

BPH, benign prostatic hyperplasia; DAPI, 4',6-diamino-2-phenylindole; GO, Gene Ontology; GSEA, gene set enrichment analysis; IGF, insulin-like growth factor; IGFBP, IGF binding protein; IL, interleukin; JAK, Janus activated kinase; KEGG, Kyoto Encyclopedia of Genes and Genomes; MMP, matrix metalloproteinase; NF-κB, nuclear factor κB; PAPPA, Pappalysin A; PTEN, phosphatase and tensin homolog; PTL, parthenolide; STAT, signal transducer and activator of transcription; TJP, tight junction protein; TMPRSS2:ERG, transmembrane protease, serine 2:v-ets erythroblastosis virus E26 oncogene homolog fusion product; TNF, tumor necrosis factor.

## Authors' contributions

RB performed microarray functional data analysis and drafted the manuscript. SDB carried out the microarray experiments and intital data analysis. CR performed the NF-κB inhibitor studies. VD carried out qRT-PCR assays. AD was involved in data analysis and design. SL performed the stem cell isolations from benign samples. PB performed stem cell isolations from tumors. CH performed culturing and isolation of stem cells, and NF-κB experiments. JLL performed the RT-PCR detection of the *TMPRSS:ERG *fusion product. MS is the surgeon who provided the patient samples, pathology results and other clinical data. NJM participated in the study design and co-ordination. AC participated in study design, analysis of experiments, writing of the manuscript and coordination of the study.
